# Hydroxylation of Platinum Surface Oxides Induced by Water Vapor

**DOI:** 10.1021/acs.jpclett.1c03927

**Published:** 2022-01-20

**Authors:** Rik V. Mom, Axel Knop-Gericke

**Affiliations:** † Fritz-Haber-Institut der Max-Plack-Gesellschaft, Faradayweg 4-6, 14195 Berlin, Germany; ‡ Leiden Institute of Chemistry, Leiden University, Einsteinweg 55, 2333 CC Leiden, The Netherlands; § Max-Planck-Institut für Chemische Energiekonversion, Stiftstrasse 34−36, 45470 Mülheim an der Ruhr, Germany

## Abstract

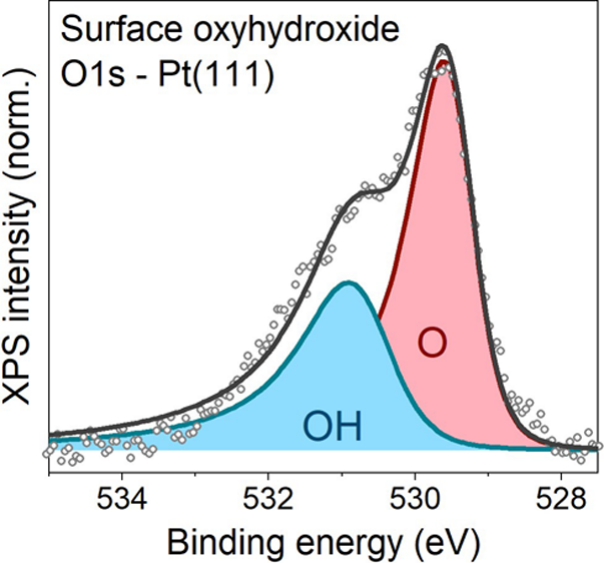

With its high stability and well-tuned binding strength for adsorbates, platinum is an excellent catalyst for a wide range of reactions. In applications like car exhaust purification, the oxidation of hydrocarbons, and fuel cells, platinum is exposed to highly oxidizing conditions, which often leads to the formation of surface oxides. To reveal the structure of these surface oxides, the oxidation of Pt in O_2_ has been widely studied. However, in most applications, H_2_O is also an important or even dominant part of the reaction mixture. Here, we investigate the interaction of H_2_O with Pt surface oxides using near-ambient-pressure X-ray photoelectron spectroscopy. We find that reversible hydroxylation readily occurs in H_2_O/O_2_ mixtures. Using time-resolved measurements, we show that O–OH exchange occurs on a time scale of seconds.

Platinum plays a central role in catalysis, both in present-day technologies such as car exhaust treatment^1^ and in future technologies such as fuel cell catalysis.^2^ Because Pt surface oxides are thought to be the catalytically active phase in many cases, knowledge of their structure is key to understanding the catalytic process. Therefore, the interaction of platinum with O_2_ has been widely investigated.^3−11^ At very low oxygen pressures, only an adsorbate overlayer is formed, with ≤0.25 monolayer (ML) coverage for the case of Pt(111).^11,12^ Starting in the range between 0.1 and 1 mbar of O_2_, the formation of oxides becomes possible.^5,8−11^ While thermodynamics predicts the onset of bulk oxidation in this pressure range (for modest temperatures),^8^ kinetic limitations ensure a wide range of stability for surface oxides up to 1 ML coverage.^3,5,9,10^ These surface oxides consist of one-dimensional chains of Pt atoms coordinated to four O atoms,^3,5,11^ following the motifs of PtO_2_ bulk oxides. Despite this structural similarity to bulk PtO_2_, the Pt atoms in the surface oxide carry very little charge, in contrast to the Pt^4+^ ions in bulk oxides.^5^ This makes the surface oxide much more reactive toward CO,^13,14^ thus confirming the belief that this surface state can participate in the catalytic cycle of oxidation reactions on platinum.

The effect of water on the structure of Pt surface oxides has not been investigated so far. This is surprising, because water generally makes up a large part of the reaction mixture in oxidation catalysis, with partial pressures often even exceeding that of O_2_. Although water itself adsorbs only weakly on clean Pt surfaces,^15^ ultra-high-vacuum studies at low temperatures have shown that water readily reacts with adsorbed oxygen, forming somewhat more stable OH adsorbates.^16,17^ To investigate whether Pt surface oxides are similarly prone to hydroxylation, we oxidized Pt(111) and a roughened Pt foil in 0.5 mbar of O_2_ at 473 K and subsequently exposed the samples to O_2_/H_2_O mixtures in the temperature range of 393–473 K (see section S1 of the Supporting Information for experimental details).

Using our NAP-XPS end-stations at the UE56-PGM1 and ISISS beamlines at the BESSY II synchrotron, we followed the chemical state of the surface through Pt 4f and O 1s spectra (details in section S2). Figure 1a shows Pt 4f spectra for the initial oxidation of the Pt(111) crystal. The asymmetric Gaussian–Lorentzian line shape used in the fitting was determined from the Pt 4f spectrum of clean Pt(111) obtained in vacuum (see section S3). Using this line shape, the fitting results for Pt surface oxides from the Nilsson group^5^ are reproduced accurately, confirming that the crystal was successfully oxidized. By comparing the O 1s/Pt 4f ratio to the 0.25 ML (2 × 2) adsorbate overlayer expected in 5 × 10^–4^ mbar of O_2_ at 463 K, we estimate the oxygen coverage to be approximately 0.5–0.6 ML after exposure to 0.5 mbar of O_2_ for 30 min.

**Figure 1:**
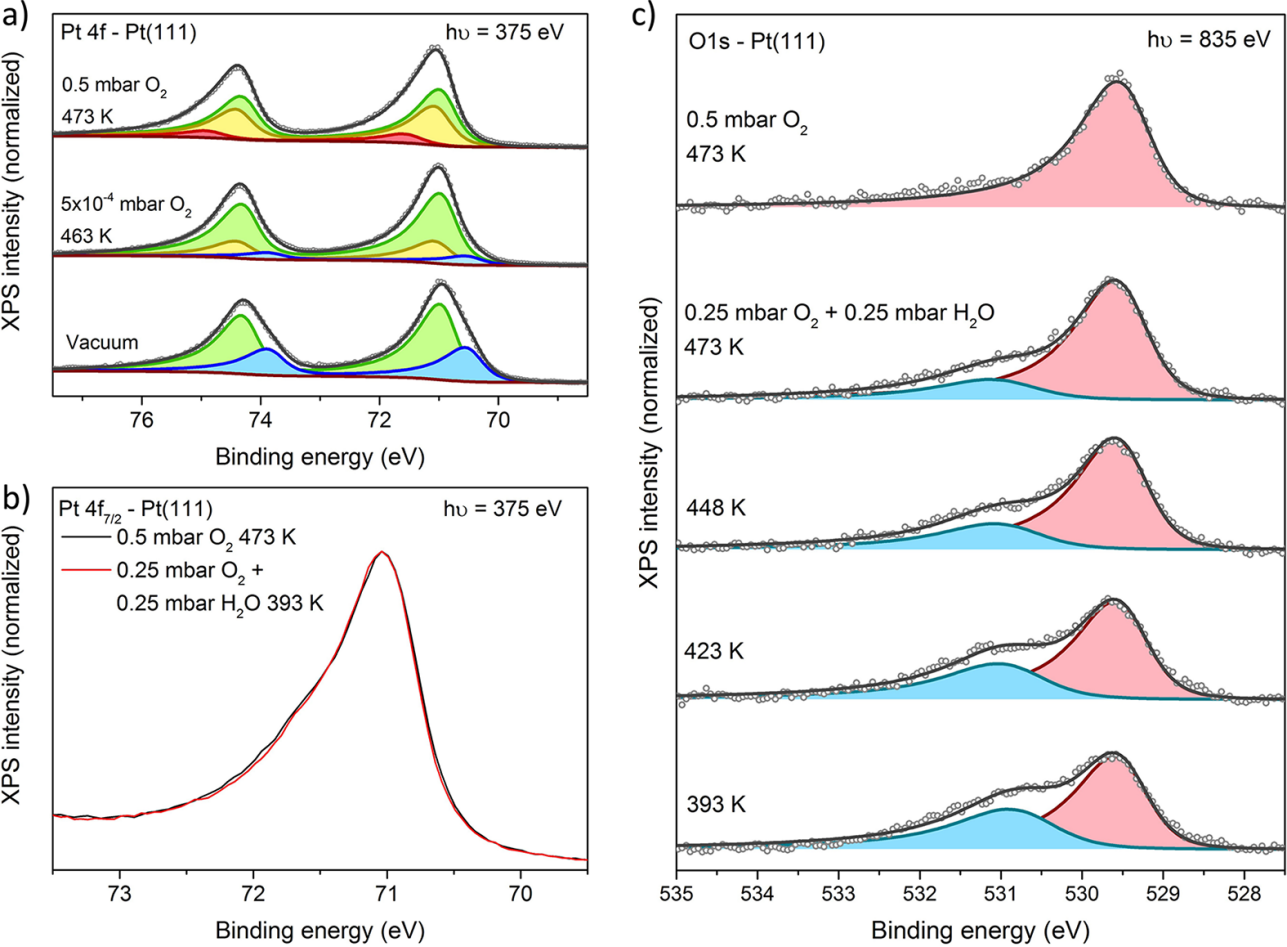
Oxidation and hydroxylation of Pt(111). (a) Initial oxidation in pure O_2_. Spectral decomposition: clean surface (blue), bulk (green), chemisorbed/surface oxide (yellow), and surface oxide (red). Fitting model from ref (5). (b) Pt 4f_7/2_ spectra before and after the introduction of H_2_O into the O_2_ environment. (c) Shirley background-subtracted O 1s spectra showing the temperature-dependent surface structure in a H_2_O/O_2_ environment. Spectral decomposition: O (red) and OH (blue).

When water is introduced into the system (0.25 mbar of H_2_O and 0.25 mbar of O_2_ at 393 K), the O 1s/Pt 4f ratio increases only 8%, indicating little to no increase in the surface coverage. This is also reflected in the Pt 4f peak shape, which is almost unchanged (see Figure 1b). However, dramatic changes are visible in the O 1s signal (Figure 1c). Under dry conditions, only a single asymmetric peak at 529.5 eV is observed, in agreement with the literature.^5,9^ The presence of water in the gas feed causes the appearance of a new peak at 530.7–531 eV. This binding energy range is typical for OH adsorbates, whereas adsorbed H_2_O would be expected at ≥532 eV.^17−19^ This assignment is further corroborated by O K-edge spectra (section S4), which display a resonance pattern consistent with an OH_ads_/O_ads_ mixture on platinum.^5,17,20^ Taking into account the fact that there is little to no change in the surface coverage, we conclude that an O–OH exchange reaction takes place:
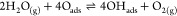
1This exchange reaction is also consistent with the observed temperature dependence of the OH peak in Figure 1c. Under the experimentally applied conditions, one can estimate the entropy change of this reaction as −0.0025 eV K^–1^ (see section S5 for a derivation). Keeping in mind the fact that the free energy of the reaction is given as Δ*G* = Δ*H* – *T*Δ*S*, this negative Δ*S* value implies that the reaction will be less favorable at an increased temperature (*T*). Indeed, Figure 1c shows that the extent of hydroxylation decreases when the temperature is increased. Note that we confirmed that the reaction can be reversed by repeatedly increasing and decreasing the temperature (see Figure S2) and that the levels of common contaminants like Si, C, and S remained below the detection limit.

To understand which sites on the Pt(111) surface become hydroxylated, it is important to consider the structural changes on the surface during the oxidation treatment prior to water exposure. Van Spronsen et al.^3^ showed that the formation of one-dimensional oxide chains is accompanied by significant surface roughening, creating approximately 10–15% step-edge sites. This number could be even higher in our case, because we performed oxidation at a slightly lower temperature (473 K here vs 526 K for van Spronsen et al.). Hence, undercoordinated sites such as step edges could provide a significant or even dominant contribution to the observed OH peak.

To investigate whether undercoordinated sites are preferentially hydroxylated, we created a defect-rich sample by prolonged Ar^+^ sputter bombardment of a Pt foil. Figure 2 shows that such a sample displays stronger hydroxylation than Pt(111), yet has a very similar temperature dependence of OH coverage. This suggests that the same hydroxylation process is occurring on the sputtered Pt foil, but that there are a larger number of favorable hydroxylation sites. Keeping in mind the large number of undercoordinated sites in the sputtered foil compared to Pt(111), we conclude that undercoordinated sites are preferentially hydroxylated.

**Figure 2:**
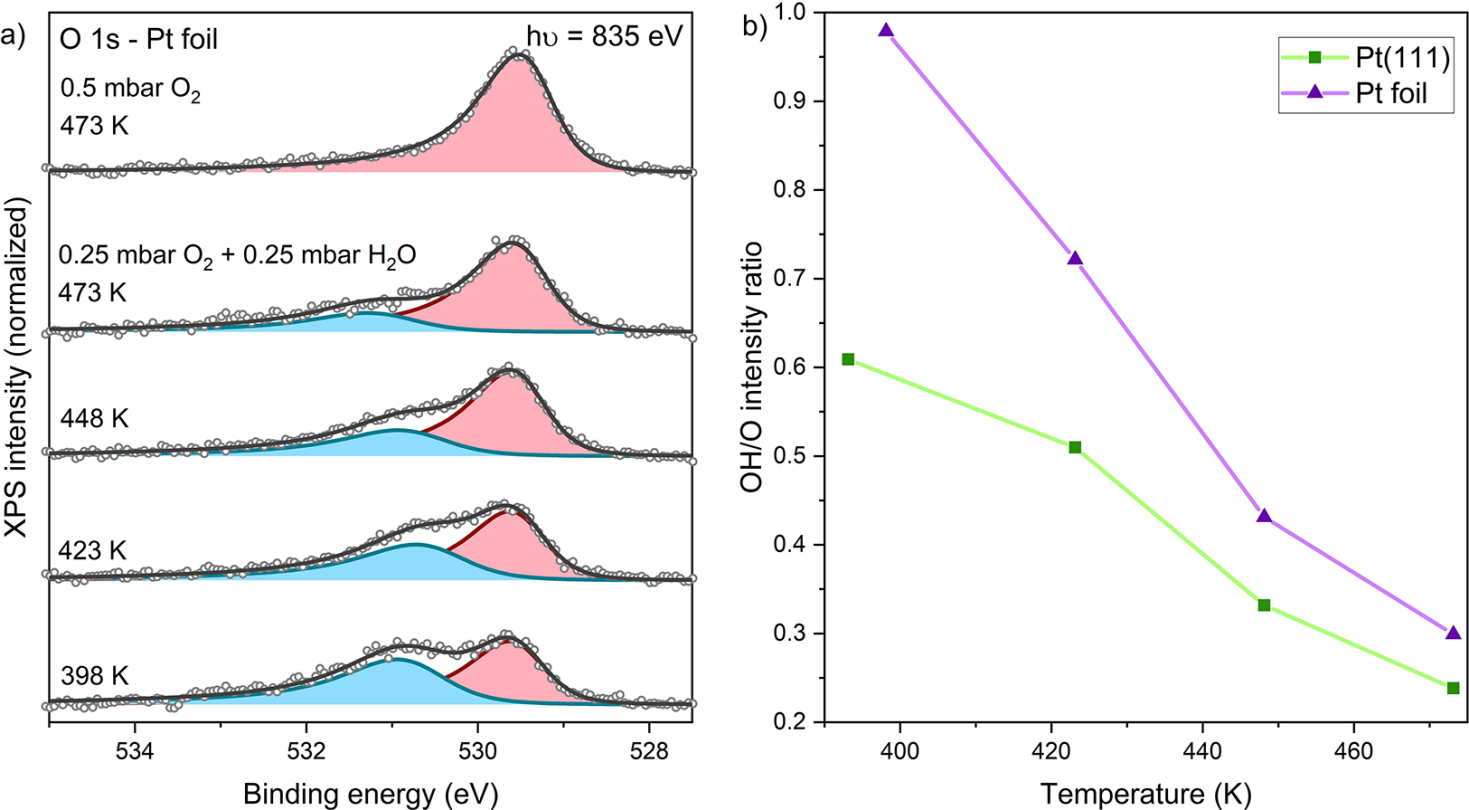
Hydroxylation of surface oxides on sputtered (defect-rich) Pt foil and comparison to Pt(111). (a) Shirley background-subtracted O 1s spectra showing the temperature-dependent surface composition in a H_2_O/O_2_ environment. Spectral decomposition: O (red) and OH (blue). (b) Peak area ratios of the OH and O peaks in Figures 1c and 2a.

Combining Figure 2b with the literature,^7^ one can obtain a rough estimate of the adsorption enthalpy of the hydroxides, approximately −1.1 eV per OH_ads_ (derivation in section S6). This is significantly higher than the value of approximately −0.9 eV for surface oxides in this coverage range,^7^ showing that the hydroxides are bound tightly to the surface. A possible explanation for this remarkably high stability could be hydrogen bonding in the hydroxide phases. However, we should also point out that the apparent adsorption enthalpy of OH could be increased by any carbon contamination in the gas phase. Although no carbon was observed on the Pt surface, traces of hydrocarbons in the gas feed would likely preferentially react with O_ads_ rather than OH_ads_, thereby increasing the observed OH_ads_/O_ads_ ratio somewhat.

Because undercoordinated sites are often considered to be the most active in catalysis, it is important to know how dynamic the hydroxylation/dehydroxylation equilibrium in eq 1 is. To test this experimentally, we performed temperature ramping experiments and followed the surface composition using O 1s spectra. Figure 3 shows that both hydroxylation and dehydroxylation occur on the time scale of a few seconds, indicating that O/OH exchange on the undercoordinated sites is very dynamic. Hence, the undercoordinated sites are regularly vacated, leaving room for catalytic turnover.

**Figure 3:**
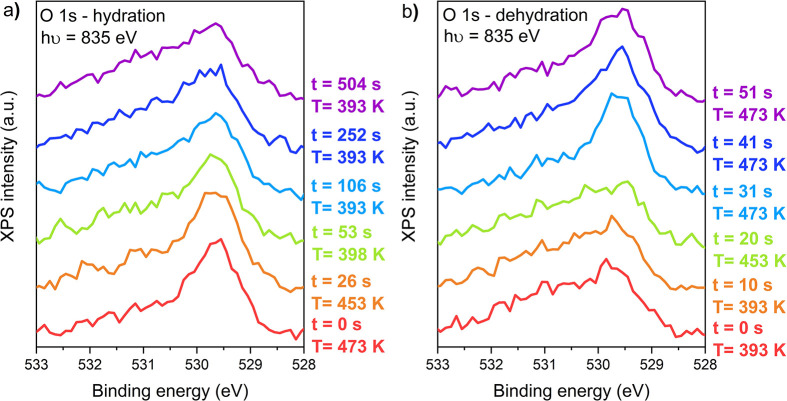
Time-resolved hydroxylation and dehydroxylation of Pt(111) in 0.25 mbar of H_2_O and 0.25 mbar of O_2_. (a) O 1s spectra while decreasing the temperature. (b) O 1s spectra while increasing the temperature. All spectra are shown Shirley background-subtracted.

In some cases, OH groups may be part of the catalytic cycle itself. To test their reactivity, we studied the catalytic combustion of hydrogen on Pt(111). As shown in Figure 4, the surface coverage of O and OH species under catalytic conditions is low, even when only trace levels of H_2_ are present in the reaction mixture. Because adsorbed OH is an intermediate of the reaction, this confirms that it is highly reactive toward hydrogen. We should point out, however, that the reactivity of the OH species may be coverage-dependent, similar to that of O species in CO oxidation.^13^

**Figure 4:**
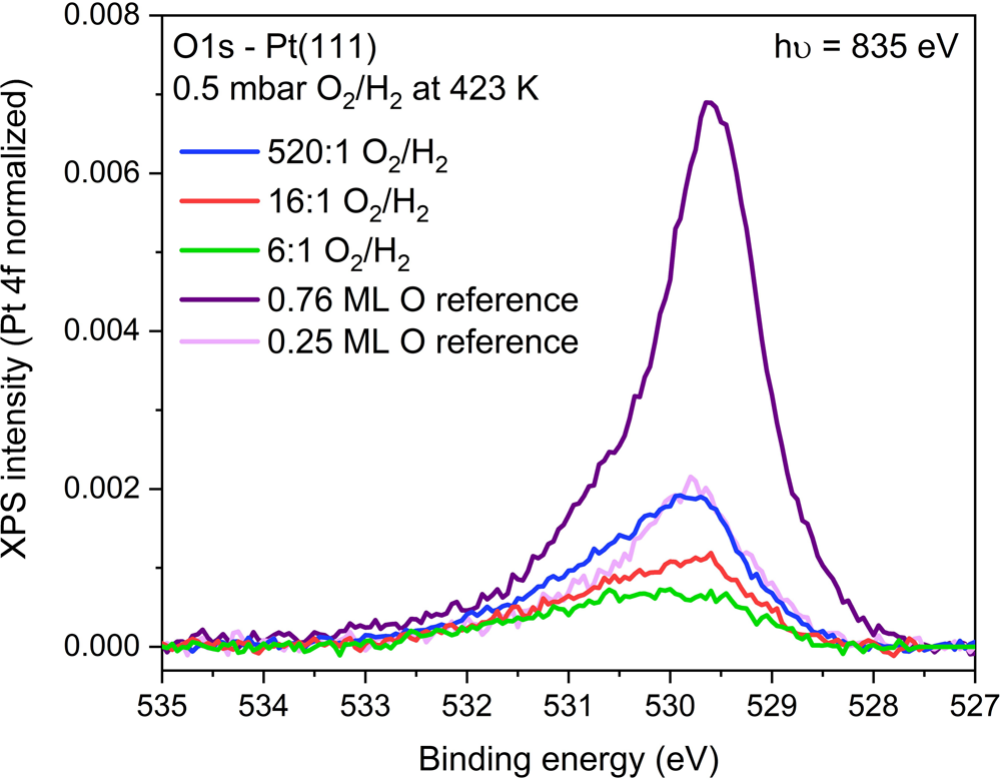
Catalytic combustion of H_2_ on Pt(111). All spectra were normalized to the Pt 4f peak area recorded with the same electron kinetic energy and are shown Shirley background-subtracted.

In conclusion, we have shown that Pt surface oxides are prone to hydroxylation, even at modest H_2_O pressures (e.g., 0.25 mbar) and elevated temperatures (393–450 K). Hence, one may expect a significant fraction of OH groups on the surface of Pt catalysts under typical oxidation catalysis conditions. The OH species replace O atoms on the surface, with a preference for undercoordinated sites. In the probed temperature range (393–473 K), the hydroxylation/dehydroxylation is highly dynamic, with a response time of a few seconds toward changes in temperature. Hence, the presence of OH sites does not irreversibly block surface sites for catalytic turnover.

## Supporting Information Available

Details on sample preparation, analysis procedures, theoretical derivations, and additional experimental data (PDF)
Transparent Peer Review report available (PDF)
