# Supplementary material for: Hydroxylation of Platinum Surface Oxides Induced by Water Vapor
Source: J Phys Chem Lett. 2022 Jan 20;13(3):879–83. doi: 10.1021/acs.jpclett.1c03927 (PMC8802315; doi:10.1021/acs.jpclett.1c03927)
Supplement: Supplementary file 1 — jz1c03927_si_001.pdf [file jz1c03927_si_001.pdf]

Supporting information

Hydroxylation of Platinum Surface Oxides Induced by Water Vapor

Rik V. Mom[1,2]*, Axel Knop-Gericke[1,3]

1. Fritz-Haber-Institut der Max-Plack-Gesellschaft, Faradayweg 4-6, 14195 Berlin, Germany
2. Leiden Institute of Chemistry, Leiden University, Einsteinweg 55, 2333 CC Leiden, The Netherlands
3. Max-Planck-Institut für Chemische Energiekonversion, Stiftstrasse 34 – 36, 45470 Mülheim an der Ruhr, Germany

*r.v.mom@lic.leidenuniv.nl

S1 Sample and gas preparation

Pt foil (ChemPUR GmbH, 99.9% purity) was cleaned by prolonged O_2 exposure at 1x10^{-4} mbar at 1000 K and cycles of 1.5 kV Ar^+ bombardment and annealing at 1100 K. The cleaning procedure was ended with 20 minutes of Ar^+ bombardment to roughen the surface. The Pt(111) crystal was cleaned by cycles of 1.5 kV Ar^+ bombardment, 1x10^{-4} mbar O_2 exposure at 1000 K and vacuum annealing at 1100 K. The cleanliness of the surface was checked at every measurement condition shown in the manuscript. Ultrapure water (Millipore) was used for the dosing experiments after degassing through freeze-pump-thaw cycles. Dosing of both H_2O, O_2, and H_2 was performed using calibrated mass flow controllers.

S2 Measurement details

The measurements were conducted at the the near-ambient pressure XPS end stations of the UE56-PGM1 and ISISS beamlines at the BESSY II/HZB synchrotron radiation facility in Berlin, Germany. The same model reaction chamber was used at both beamlines. XPS measurements were performed with a pass energy of 10 eV. For Pt 4f spectra, a reduced exit slit of 20 micrometer was set to improve the energy resolution, whereas 180 micrometer was used for the other spectra. The incoming X-rays had an incidence angle of 34° and were linearly polarized in the plane perpendicular to the surface. Photoelectrons were collected along the surface normal.

To achieve suppression of gas phase signals in the O K-edge XAS spectra, we made use of auger electron yield (AEY) detection to measure the absorption. Using the electron analyzer, we selectively detect Auger electrons with a kinetic energy of 514.4 eV. This coincides with one of the Auger O KLL peaks of the surface species on Pt. In contrast, gas phase O_2 and H_2O do not have a O KLL peak at this energy[1,2]. Consequently, the gas phase signal does not appear in the Auger-yield XAS O K-edge spectrum.

The experimental resolution for the O 1s measurements was about 450 meV (based on Xe $5p_{3/2}$ measurements). For the O K-edge spectra, the resolution was about 220 meV. For the Pt 4f spectra, the resolution was about 200 meV.

S3 Data analysis

Background subtraction and peak fitting of the measured spectra was performed using the CasaXPS 2.3.18PR1.0 software package[3]. The O 1s and Pt 4f spectra were fitted using the settings listed in Tables S1 and S2.

Table S1: Fitting parameters for O 1s spectra

	**O**	**OH**
Line shape	LF(0.8, 2, 50, 70)	LF(0.8, 2, 50, 70)
Peak position	529.5-529.6 eV	Free
FWHM	0.92 eV	1.25-1.29 eV

Table S2: Fitting parameters for Pt 4f spectra

	**Pt^0**	**Pt-surf**	**Pt-chem**	**Pt-4O**
Line shape	LF(0.75,1.8,30, 40)	LF(0.75,1.8,30, 40)	LF(0.75,1.8,30, 40)	LF(0.75,1.8,30, 40)
Peak position $4f_{7/2}$	70.96 eV	70.53 eV	71.05 eV	71.05-72 eV
FWHM	0.67 eV	0.67 eV	0.67 eV	0.67 eV
Spin orbit splitting	3.34 eV	3.34 eV	3.34 eV	3.34 eV

The LF line shape chosen here is an asymmetric gaussian-lorentzian line shape. This type of line shape was chosen to accommodate the asymmetry in the both the O1s and Pt 4f peaks, which is often observed for metallic surfaces. It results from the electron-hole pair excitations that occur along with the photo-emission of the O 1s or Pt 4f electron, and is quite pronounced here due to the high density of states around the Fermi level for Pt. Several asymmetric line shapes can be used to describe the asymmetry, all of which are somewhat empirical. We choose the LF line shape here because it has a fairly flexible shape that can also capture instrumental broadening, and because its tail towards higher binding energy is relatively short. The latter point is important to have an unambiguous fit, since one is able to separate what is peak and what is background (this is a problem for the Doniach-Sunjic line shape, for example).

The LF line shape is parameterized by four parameters: α, β, γ, and w, overall expressed as LF(α,β,γ,w). γ is the width of the gaussian with which the lorentzian is convoluted. The combination of α and β determines the asymmetry of the peak. Finally, w is controls the dampening of the tail of the peak. A full mathematical description of the line shape can be found in ref[4].

The values of the line shape parameters for the Pt 4f spectra were determined using clean Pt(111) in high vacuum (~10^{-8} mbar) at ~820 K. This high temperature was chosen to prevent the adsorption of trace gases present in the vacuum chamber. In addition, the spectrum was recorded directly after cleaning. The Pt 4f spectrum of clean Pt(111) contains two components: a bulk and a surface component. The binding energy of the surface component lies about 0.43 eV below that the bulk component[5] and based on the in elastic mean free path of photoelectrons with the chosen 300 eV kinetic energy, one expects a peak ratio of roughly 0.45[6]. With our optimized line shape, we obtained a value of 0.4 for the peak ratio, which is fairly accurate also taking into account that a small amount of adsorbates on the surface cannot be excluded. For the O1s spectra, the line shape was determined in 0.5 mbar O_2 at 473 K, which generates a single component in the O 1s spectrum[5] that can be used as a direct measurement of the line shape.

During the fitting of the O 1s spectra, the FWHM of the O-component was kept fixed to its value determined in pure O_2. The other parameters were left free, because the binding energy of the O and OH components likely depends on the coverage of both O and OH.

To fit the Pt 4f spectra, we based ourselves on the fitting model used by Miller *et al.* for metallic and surface oxidized Pt[5]. The model has four contributions: Pt^0 (bulk metallic Pt), Pt-surf (metallic Pt on the surface without adsorbates), Pt-chem (metallic Pt on the surface with adsorbed O on it), and Pt-4O (Pt in a surface oxide). The binding energies for each component were determined using a more extensive dataset than the one presented in Figure 1a here. The binding energies of the Pt^0, Pt-surf, and Pt-chem components also proved consistent with earlier work and studies of other Pt single crystal surfaces.

During the fitting of our Pt 4f spectra, the FWHM and spin-orbit splitting were kept constant for all components in all spectra (always 0.67 eV and 3.34 eV, respectively). The peak areas of the two spin-orbit peaks of each fitting component were forced to obey the 4:3 ratio expected for a $4f_{7/2}/4f_{5/2}$ pair. The peak positions of the Pt^0, Pt-surf, and Pt-chem components were determined iteratively, so that one peak position was determined for all spectra (e.g. 70.96 eV for the $4f_{7/2}$ peak of Pt^0 in all three spectra).

S4 O K-edge spectra

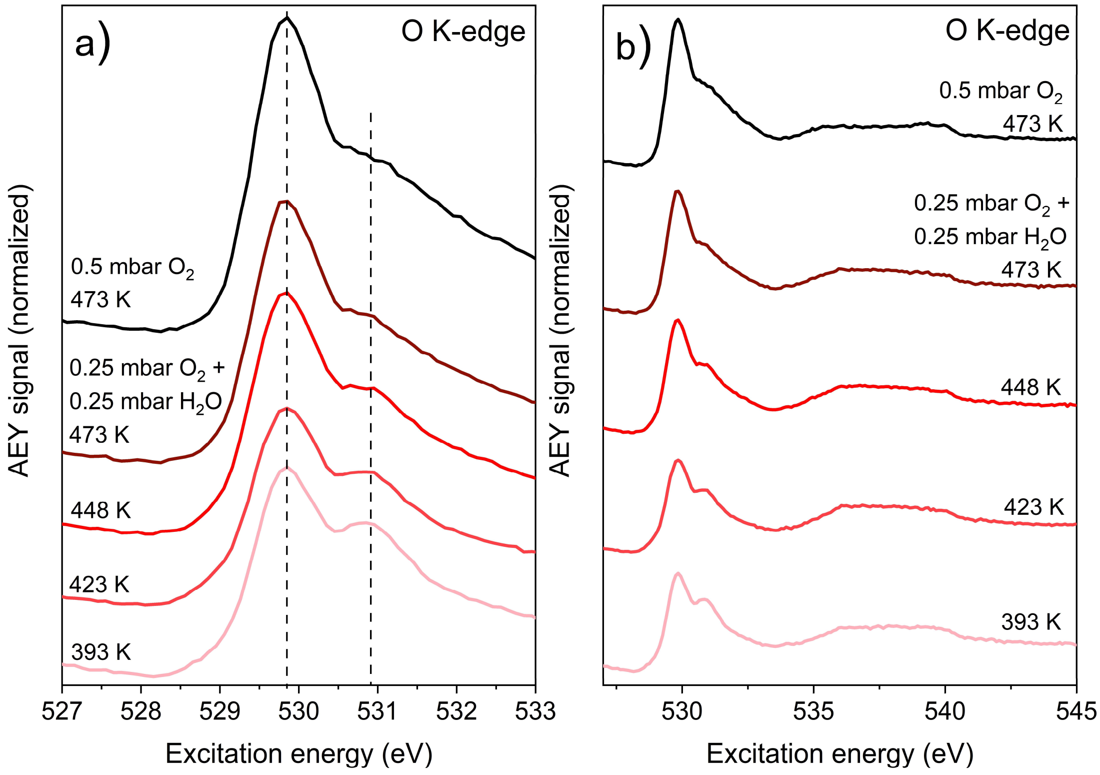

Figure S1: O K-edge spectra of Pt(111) in various conditions.

The O K-edge spectra corroborate the assignments given in the main text. In a pure O_2 atmosphere, a strong resonance at 529.8 eV is observed with a broad, weak shoulder at ~531.1 eV. This was observed previously for Pt surface oxides (without OH groups) in XAS experiments that employed the same X-ray polarization direction as done here[5]. When water is introduced into the chamber and the temperature is decreased, a resonance at 530.9 eV develops. This is resonance energy is lower than for common contaminants such as hydrocarbons[7] and silicon oxide[8]. Indeed, in initial measurements with a lower level of cleanliness, the hydrocarbon resonance was observed at ~532 eV. On the other

hand, the resonance at 530.9 eV fits well with the energy range from literature reports on OH groups on the surface of Pt[9,10].

Note that the gas phase signals from O_2 and H_2O were suppressed by the choice of kinetic energy in the Auger electron yield (AEY) detection mode (see Section S2 for details).

S5 Derivation of entropy change

Here, we derive the entropy change of the reaction:

$$2H_2O_{(g)} + 4O_{ads} \rightleftharpoons 4OH_{ads} + O_{2(g)} \quad \text{(S1)}$$

The entropies of gas phase species are readily found in standard tables[11], whereas the entropy of adsorbates is usually small so that they can be neglected[12,13]. With this approximation, the adsorption entropy for the reaction in equation S1 can be calculated as:

$$\Delta S_{hydro} \approx S^o_{O_2} - 2S^o_{H_2O} - R\ln\left(\frac{P_{O_2}}{P^o}\right) + 2R\ln\left(\frac{P_{H_2O}}{P^o}\right) = 205.15\frac{J}{molK} - 377.68\frac{J}{molK} + 68.957\frac{J}{molK} - 137.91\frac{J}{molK} = -241.48\frac{J}{molK} = -0.0025\ eV\ K^{-1} \quad \text{(S2)}$$

In equation S2, the S^o_{O2} and S^o_{H2O} designate the standard entropies of oxygen and water, respectively. R is the gas constant, P_{O2} is the oxygen pressure, P_{H2O} is the water pressure and P^o is the standard pressure for which the standard entropies were defined (1 bar). The third and fourth term in equation S2 account for the difference between the experimental partial pressures (0.25 mbar for both O_2 and H_2O) and standard pressure (1 bar).

S6 Derivation of adsorption enthalpy estimate

First, we note that the free energy change of reaction S1 is calculated as:

$$\Delta G_{hydro} = \Delta H_{hydr} - T\Delta S_{hydrox} \quad \text{(S3)}$$

If we assume that $\Delta G_{hydrox} = 0$ at the OH coverage inflection point in Figure 2b in the main text, then:

$$\Delta H_{hydro} = T\Delta S_{hydrox} = 435 \cdot -0.0025 = -1.08\ eV\ per\ O_2 = -0.27\ eV\ per\ OH_{ads} \quad \text{(S4)}$$

Reaction S1 is not an adsorption reaction, but rather a conversion from O_{ads} to OH_{ads}. Hence, ΔH_{hydrox} is only a measure of the relative stability of surface hydroxides with respect to surface oxides. To estimate the hydroxide adsorption energy, we consider the adsorption reactions:

$$O_{2(g)} \rightleftharpoons 2O_{ads} \quad \text{(S5)}$$

$$2H_2O_{(g)} + O_{2(g)} \rightleftharpoons 4OH_{ads} \quad \text{(S6)}$$

Note that reaction S1 = reaction S6 - reaction S5. From this, it follows that:

$$\Delta H_{hydrox} = \Delta H_{OHads} - \Delta H_{Oads} \quad \text{(S7)}$$

The adsorption energy of surface oxides has been calculated using *ab initio* methods for various structures. Based on the experimentally applied conditions and the observed coverage (0.5-0.6 ML) in our experiments, the representative surface oxide structures at the step edges have an adsorption energy ΔH_{Oads} of about -0.8 eV to -0.9 eV per O_{ads}[14]. Based on this, the adsorption enthalpy of the hydroxides ΔH_{OHads} is estimated to be -1.1 eV to -1.2 eV per OH_{ads}.

S7 Reversibility of the hydroxylation of surface oxides on Pt(111)

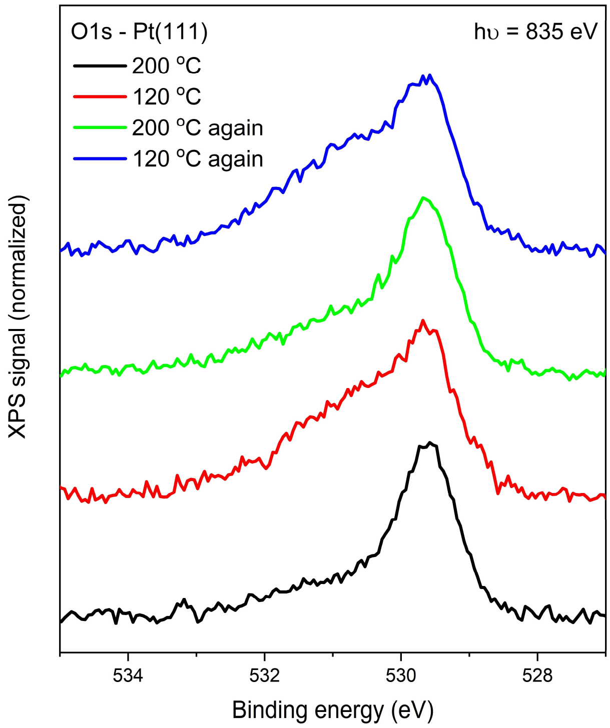

Figure S2: Shirley background-subtracted O1s spectra showing the reversible hydroxylation of surface oxides on Pt(111). All spectra were recorded in 0.25 mbar H_2O + 0.25 mbar O_2.

To investigate whether the observed changes in the O1s spectra of Pt surface oxides on Pt(111) are reversible, we cycled the temperature up and down multiple times. The O1s spectra in Figure S1 show that this procedure yields reversible hydroxylation and dehydroxylation. This also confirms that there was little to no build-up of oxygen-containing impurities such as SiO_x or hydrocarbons, in agreement with the absence of peaks in the Si2p and C1s regions. Note that the data in Figure 1c in the main text was recorded between the red and the green curve in Figure S1.

References

(1) Hävecker, M.; Cavalleri, M.; Herbert, R.; Follath, R.; Knop-gericke, A.; Hess, C.; Hermann, K.; Schlögl, R. Methodology for the Structural Characterization of VxOy Species Supported on Silica under Reaction Conditions by Means of in Situ O K-Edge X-Ray Absorption Spectroscopy. *Phys. Stat. Sol.* **2009**, *246* (7), 1459–1469. https://doi.org/10.1002/pssb.200945067.

(2) Patel, D. I.; Shah, D.; Bahr, S.; Dietrich, P.; Meyer, M.; Thißen, A.; Linford, M. R. Water Vapor, by near-Ambient Pressure XPS. *Surf. Sci. Spectra* **2019**, *26* (1), 014026. https://doi.org/10.1116/1.5111634.

(3) Fairley, N. CasaXPS 2.3. 1999.

(4) Major, G. H.; Shah, D.; Avval, T. G.; Fernandez, V.; Fairley, N.; Linford, M. R.; Rouxel, J. Advanced Line Shapes in X-Ray Photoelectron Spectroscopy II.The Finite Lorentzian (LF). *Vac. Technol. Coat.* **2020**, No. 4, 35–39.

(5) Miller, D. J.; Öberg, H.; Kaya, S.; Sanchez Casalongue, H.; Friebel, D.; Anniyev, T.; Ogasawara, H.; Bluhm, H.; Pettersson, L. G. M.; Nilsson, A. Oxidation of Pt(111) under near-Ambient Conditions. *Phys. Rev. Lett.* **2011**, *107*, 195502. https://doi.org/10.1103/PhysRevLett.107.195502.

(6) Cumpson, P. J.; Seah, M. P. Elastic Scattering Corrections in AES and XPS. II. Estimating Attenuation Lengths and Conditions Required for Their Valid Use in Overlayer/Substrate Experiments. *Surf. Interface Anal.* **1997**, *25* (6), 430–446. https://doi.org/10.1002/(SICI)1096-9918(199706)25:6<430::AID-SIA254>3.0.CO;2-7.

(7) Prince, K. C.; Richter, R.; De Simone, M.; Alagia, M.; Coreno, M. Near Edge X-Ray Absorption Spectra of Some Small Polyatomic Molecules. *J. Phys. Chem. A* **2003**, *107*, 1955–1963. https://doi.org/10.1021/jp0219045.

(8) Wallis, D. J.; Gaskell, P. H.; Brydson, R. Oxygen K Near-edge Spectra of Amorphous Silicon Suboxides. *J. Microsc.* **1995**, *180* (3), 307–312. https://doi.org/10.1111/j.1365-2818.1995.tb03690.x.

(9) Nagasaka, M.; Nakai, I.; Kondoh, H.; Ohta, T.; Carravetta, V. Oxygen K-Edge near Edge X-Ray Absorption Fine Structures of O and OH Overlayers on Pt(1 1 1). *Chem. Phys. Lett.* **2003**, *375* (3–4), 419–424. https://doi.org/10.1016/S0009-2614(03)00876-5.

(10) Schiros, T.; Näslund, L.-Å.; Andersson, K.; Gyllenpalm, J.; Karlberg, G. S.; Odelius, M.; Ogasawara, H.; Pettersson, L. G. M.; Nilsson, A. Structure and Bonding of the Water-Hydroxyl Mixed Phase on Pt(111). *J. Phys. Chem. C* **2007**, *111*, 15003–15012. https://doi.org/10.1021/jp073405f.

(11) NIST. Computational Chemistry Comparison and Benchmark Database http://cccbdb.nist.gov/.

(12) Shi, H.; Stampfl, C. First-Principles Investigations of the Structure and Stability of Oxygen Adsorption and Surface Oxide Formation at Au(111). *Phys. Rev. B* **2007**, *76* (7), 075327. https://doi.org/10.1103/PhysRevB.76.075327.

(13) Lauritsen, J. V.; Bollinger, M. V.; Lægsgaard, E.; Jacobsen, K. W.; Nørskov, J. K.; Clausen, B. S.; Topsøe, H.; Besenbacher, F. Atomic-Scale Insight into Structure and Morphology Changes of MoS2 Nanoclusters in Hydrotreating Catalysts. *J. Catal.* **2004**, *221* (2), 510–522. https://doi.org/10.1016/j.jcat.2003.09.015.

(14) Bandlow, J.; Kaghazchi, P.; Jacob, T.; Papp, C.; Tränkenschuh, B.; Streber, R.; Lorenz, M. P. A.; Fuhrmann, T.; Denecke, R.; Steinrück, H. P. Oxidation of Stepped Pt(111) Studied by x-Ray Photoelectron Spectroscopy and Density Functional Theory. *Phys. Rev. B - Condens. Matter Mater. Phys.* **2011**, *83*, 1–5. https://doi.org/10.1103/PhysRevB.83.174107.
