# Supplementary material for: Hydroxylation of Platinum Surface Oxides Induced by Water Vapor
Source: J Phys Chem Lett. 2022 Jan 20;13(3):879–83. doi: 10.1021/acs.jpclett.1c03927 (PMC8802315; doi:10.1021/acs.jpclett.1c03927)
Supplement: Supplementary file 2 — jz1c03927_si_002.pdf [file jz1c03927_si_002.pdf]

jz-2021-03927s.R1

Name: Peer Review Information for "The Hydroxylation of Platinum Surface Oxides Induced by Water Vapor"

First Round of Reviewer Comments

Reviewer: 1

Comments to the Author
Report on “The hydroxylation of platinum surface oxides induced by water vapor” by Mom et al.

The paper is a well written article on the hydroxylation of PtOx, studied by synchrotron based near ambient pressure XPS. The paper should be well suited for Journal of Physical Chemistry Letters after minor revisions. Details are given below.
The discussion of the exchange reaction (hydroxylation / dehydroxylation) of the oxide is quite interesting. Could one also argue along the lines of the desorption temperature of either H2 or H2O?
Please add a fit to the data in Fig. 3. This would allow for a better understanding of the changes during reaction.
Please add the experimental resolution of the measurements to the SI

Reviewer: 2

Comments to the Author
This manuscript is focussed on an interesting topic, the humid oxidation of Pt, which would appeal to both the heterogeneous catalysis and the electrochemistry communities. It has the potential to become an influential paper. However, I believe the following changes are required before the paper can be considered for publication in JPCL:

1. Comparing the results obtained from a sputtered polycrystalline foil with those from a well-annealed single crystal to correlate activity with defect sites is not justified. In addition to defects, the sputtered foil will also contain non-111 surfaces with lower coordinated Pt atoms, which may explain the difference in reactivity between the foil and the single crystal.

The comparison that should be made is either between a sputtered and non-sputtered single crystal or between a sputtered and non-sputtered foil. Without these additional experiments, the increased reactivity cannot be unequivocally assigned to defects and the claim should be reduced to “lower coordinated Pt atoms are more reactive” (which is not very surprising).

Quantification of the increased density of defects induced by the sputtering using SPM would further strengthen this argument. Even more so, because the oxidation itself roughens the surface.

2. The Pt4f fits are not very convincing. This is mostly because crucial information is missing

a. The fitting function should be given. The label "LF" is insufficient as the paper should be understandable without having to resort to the CasaXPS manual. Furthermore, the different fit parameters need to be clarified and for every fit it needs to be specified, which of them were free fitting parameters and which were constraint. Justification for constrains are also important. This is critical since the fitting function seems to contain 6 fitting parameters per peak. Finally, a motivation is required for the choice of this empirical function.

b. The presence of the clean surface Pt peak needs further supporting. Typically, the base pressure of NAP XPS systems is not that great and the presence of a fully empty surface may not be likely. At least, surface temperature and pressure need to be stated. Showing survey and C1s spectra would further enhance the reliability of this claim. At this given KE/IMFP is the surface-bulk ratio what you would expect?

c. The binding energy difference between bulk Pt and Pt-chem is only 0.09 eV, which is a small shift. In this light, I wonder how much the choice of the fitting function influences the outcome of this fitting model. This feeling is strengthened by the fact that the binding energy of the Pt-4O has a range of about 1 eV, which is ten times the shift of the Pt-chem peak with respect to the Pt bulk peak. All together, the results seem quite arbitrary. I realize given the fact that the Pt 4f spectra all look very similar, this is somewhat expected. Therefore, it would be good to link the intensity of the Pt-chem and Pt-4O peaks with their corresponding O1s counterparts to at least show that there is internal consistency in the fitting model.

3. "However, dramatic changes are visible in the O1s signal" Looking at Fig 1c, "dramatic" does not seem the right word here as all the O1s spectra of Fig 1c are not that different.

4. XAS spectra, important information is missing. How where these obtained (transmission, TEY, TFY)? Why are the spectral features of the gas phase not visible in these spectra? Similarly, why are the gas phase peaks not visible in the XPS spectra?

None of these points are trivial and deserve careful discussion and possibly some additional experiments.

Author's Response to Peer Review Comments:

Dear EDITOR,

We are pleased to note that the reviewers are positive about the manuscript, noting that "it has the potential to become an influential paper" and that it "should be well suited for Journal of Physical Chemistry Letters after minor revisions". We thank the reviewers for their comments, which have helped to improve the manuscript. With the revisions outlined below, we believe that the manuscript is now suitable for publication in JPCL.

With kind regards,

Rik Mom

Reviewer: 1

Recommendation: This paper is publishable subject to minor revisions noted. Further review is not needed.

Comments:
Report on “The hydroxylation of platinum surface oxides induced by water vapor” by Mom et al.

The paper is a well written article on the hydroxylation of PtOx, studied by synchrotron based near ambient pressure XPS. The paper should be well suited for Journal of Physical Chemistry Letters after minor revisions. Details are given below.

The discussion of the exchange reaction (hydroxylation / dehydroxylation) of the oxide is quite interesting. Could one also argue along the lines of the desorption temperature of either H2 or H2O?

Our initial interpretation went along the same line as the reviewer, that H2O adsorbs dissociatively on the oxide. However, as we note in the manuscript, the O coverage remains constant when water is introduced into the chamber. This does not fit with an adsorption reaction, where the coverage should increase. Rather, there is a replacement of adsorbates (Reaction 1 in the manuscript).

To describe dehydroxylation in terms of H2 desorption, one would assume the reaction:

$2\ OH_{ads} \rightarrow 2\ O_{ads} + H_2$

Since there is essentially no H_2 in the gas phase of the chamber, this would be an irreversible reaction. However, Figures 3 and S7 show that the reaction is reversible.

Hence, we conclude that discussing the hydroxylation/dehydroxylation behavior in terms of a desorption temperature would potentially set the reader on the wrong foot.

Please add a fit to the data in Fig. 3. This would allow for a better understanding of the changes during reaction.

Although we agree with the reviewer that a fit can be helpful for a more quantitative understanding of the changes of the surface state, we should note that the time-resolved measurements inherently had a lower data quality. Not only is the signal-to-noise ratio lower than for the steady-state spectra, we also had to employ a smaller binding energy range to decrease the acquisition time, making the baseline for the fit somewhat ambiguous. For these reasons, we feel that a fit of the data would not be sufficiently reliable and could potentially misguide the reader.

Please add the experimental resolution of the measurements to the SI

We have indicated the experimental resolution in SI Section S2:

The experimental resolution for the O 1s measurements was about 450 meV (based on Xe 5p$_{3/2}$ measurements). For the O K-edge spectra, the resolution was about 220 meV. For the Pt 4f spectra, the resolution was about 200 meV.

Reviewer: 2

Recommendation: This paper may be publishable, but major revision is needed; I would like to be invited to review any future revision.

Comments:
This manuscript is focussed on an interesting topic, the humid oxidation of Pt, which would appeal to both the heterogeneous catalysis and the electrochemistry communities. It has the potential to become an influential paper. However, I believe the following changes are required before the paper can be considered for publication in JPCL:

1. Comparing the results obtained from a sputtered polycrystalline foil with those from a well-annealed single crystal to correlate activity with defect sites is not justified. In addition to defects, the sputtered foil will also contain non-111 surfaces with lower coordinated Pt atoms, which may explain the difference in reactivity between the foil and the single crystal.
The comparison that should be made is either between a sputtered and non-sputtered single crystal or between a sputtered and non-sputtered foil. Without these additional experiments, the increased reactivity cannot be unequivocally assigned to defects and the claim should be reduced to "lower coordinated Pt atoms are more reactive" (which is not very surprising).

Quantification of the increased density of defects induced by the sputtering using SPM would further strengthen this argument. Even more so, because the oxidation itself roughens the surface.

We thank the reviewer for this comment, which has indeed also been discussed internally. We considered the term "defects" as sufficiently general to highlight the structural differences between the sputtered foil and the single crystal. However, we agree that the term "lower-coordinated Pt atoms" is more suitable, as it more clearly includes other facets. We have replaced the term "defect sites" in the manuscript.

We should point out that the revised statement is not a trivial case of "lower coordinated Pt atoms are more reactive", since we are exchanging one adsorbate for another here. Hence, it is not about the reactivity of the lower coordinated sites, but rather the selectivity for the O_{ads} or OH_{ads} adsorbate. The fact that lower coordinated sites are more selective towards OH_{ads} than (111) terraces does not seem trivial.

2. The Pt4f fits are not very convincing. This is mostly because crucial information is missing
a. The fitting function should be given. The label "LF" is insufficient as the paper should be understandable without having to resort to the CasaXPS manual. Furthermore, the different fit parameters need to be clarified and for every fit it needs to be specified, which of them were free fitting parameters and which were constraint. Justification for constrains are also important. This is critical since the fitting function seems to contain 6 fitting parameters per peak. Finally, a motivation is required for the choice of this empirical function. b. The presence of the clean surface Pt peak needs further supporting. Typically, the base pressure of NAP XPS systems is not that great and the presence of a fully empty surface may not be likely. At least, surface temperature and pressure need to be stated. Showing survey and C1s spectra would further enhance the reliability of this claim. At this given KE/IMFP is the surface-bulk ratio what you would expect?

c. The binding energy difference between bulk Pt and Pt-chem is only 0.09 eV, which is a small shift. In this light, I wonder how much the choice of the fitting function influences the outcome of this fitting model. This feeling is strengthened by the fact that the binding energy of the Pt-4O has a range of about 1 eV, which is ten times the shift of the Pt-chem peak with respect to the Pt bulk peak. All together, the results seem quite arbitrary. I realize given the fact that the Pt 4f spectra all look very similar, this is somewhat expected. Therefore, it would be good to link the intensity of the Pt-chem and Pt-4O peaks with their corresponding O1s counterparts to at least show that there is internal consistency in the fitting model.

The reviewer raises some just points about the fitting model. We agree that more details could be helpful for the reader. Therefore, we have added a detailed description of the fitting model and our choices in the SI (copied below for your convenience). A few points we should note:

- The mathematical form of the LF line shape is rather complex. Therefore, we chose to explain the parameters rather than show the function and provided a reference to a more detailed treatment for the interested reader (the CasaXPS manual actually does not show the full function).
- The Pt 4f fitting model was based on the model of Miller et al. and is merely used to check the consistency of our data with previously published results. The Pt 4f data in Figure 1a is not used to derive any new conclusions here. Nonetheless, we have justified the choices in our fit model.

New text in SI section S3:

The LF line shape chosen here is an asymmetric gaussian-lorentzian line shape. This type of line shape was chosen to accommodate the asymmetry in the both the O1s and Pt 4f peaks, which is often observed for metallic surfaces. It results from the electron-hole pair excitations that occur along with the photo-emission of the O 1s or Pt 4f electron, and is quite pronounced here due to the high density of states around the Fermi level for Pt. Several asymmetric line shapes can be used to describe the asymmetry, all of which are somewhat empirical. We choose the LF line shape here because it has a fairly flexible shape that can also capture instrumental broadening, and because its tail towards higher binding energy is relatively short. The latter point is important to have an unambiguous fit, since one is able to separate what is peak and what is background (this is a problem for the Doniach-Sunjic line shape, for example).

The LF line shape is parameterized by four parameters: α, β, γ, and w, overall expressed as LF(α,β,γ,w). γ is the width of the gaussian with which the lorentzian is convoluted. The combination of α and β determines the asymmetry of the peak. Finally, w is controls the dampening of the tail of the peak. A full mathematical description of the line shape can be found in ref[2].

The values of the line shape parameters for the Pt 4f spectra were determined using clean Pt(111) in high vacuum (~10^{-8} mbar) at ~820 K. This high temperature was chosen to prevent the adsorption of trace gases present in the vacuum chamber. In addition, the spectrum was recorded directly after cleaning. The Pt 4f spectrum of clean Pt(111) contains two components: a bulk and a surface component. The binding energy of the surface component lies about 0.43 eV below that the bulk component[3] and based on the in elastic mean free path of photoelectrons with the chosen 300 eV kinetic energy, one expects a peak ratio of roughly 0.45[4]. With our optimized line shape, we obtained a value of 0.4 for the peak ratio, which is fairly accurate also taking into account that a small amount of adsorbates on the surface cannot be excluded. For the O1s spectra, the line shape was determined in 0.5 mbar O_2 at 473 K, which generates a single component in the O 1s spectrum[3] that can be used as a direct measurement of the line shape.

During the fitting of the O 1s spectra, the FWHM of the O-component was kept fixed to its value determined in pure O_2. The other parameters were left free, because the binding energy of the O and OH components likely depends on the coverage of both O and OH.

To fit the Pt 4f spectra, we based ourselves on the fitting model used by Miller et al. for metallic and surface oxidized Pt[3]. The model has four contributions: Pt^0 (bulk metallic Pt), Pt-surf (metallic Pt on the surface without adsorbates), Pt-chem (metallic Pt on the surface with adsorbed O on it), and Pt-4O (Pt in a surface oxide). The binding energies for each component were determined using a more extensive dataset than the one presented in Figure 1a here. The binding energies of the Pt^0, Pt-surf, and Pt-chem components also proved consistent with earlier work and studies of other Pt single crystal surfaces.

During the fitting of our Pt 4f spectra, the FWHM and spin-orbit splitting were kept constant for all components in all spectra (always 0.67 eV and 3.34 eV, respectively). The peak areas of the two spin-orbit peaks of each fitting component were forced to obey the 4:3 ratio expected for a $4f_{7/2}/4f_{5/2}$ pair. The peak positions of the Pt^0, Pt-surf, and Pt-chem components were determined iteratively, so that one peak position was determined for all spectra (e.g. 70.96 eV for the $4f_{7/2}$ peak of Pt^0 in all three spectra).

3. “However, dramatic changes are visible in the O1s signal” Looking at Fig 1c, “dramatic” does not seem the right word here as all the O1s spectra of Fig 1c are not that different.

This is a bit of a subjective issue, of course. In our opinion, the changes look rather significant (we were surprised). The word dramatic is an expression of our surprise, which we think may be shared by many readers.

4. XAS spectra, important information is missing. How where these obtained (transmission, TEY, TFY)? Why are the spectral features of the gas phase not visible in these spectra? Similarly, why are the gas phase peaks not visible in the XPS spectra?

We thank the reviewer for raising this point, we agree that we forgot to mention some important details here.

In SI Section S2, we now added:

To achieve suppression of gas phase signals in the O K-edge XAS spectra, we made use of auger electron yield (AEY) detection to measure the absorption. Using the electron analyzer, we selectively detect Auger electrons with a kinetic energy of 514.4 eV. This coincides with one of the Auger O KLL peaks of the surface species on Pt. In contrast, gas phase O_2 and H_2O do not have a O KLL peak at this energy[1,2]. Consequently, the gas phase signal does not appear in the Auger-yield XAS O K-edge spectrum.

In SI Section S4, we added:

Note that the gas phase signals from O_2 and H_2O were suppressed by the choice of kinetic energy in the Auger electron yield (AEY) detection mode (see Section S2 for details).

For the gas phase O 1s peaks, we did not add further details because these peaks fall outside the spectral region that we show in the manuscript (536 eV for water vapor, 540 eV for gas phase O_2). However, note that we apply a bias to the analyzer cone that shifts/broadens the gas phase peaks, which suppresses the gas phase contribution. As a result, it does not show up in the spectra (we did record up to 538 eV).
